# The Isolation and Characterization of Novel *Caulobacter* and Non-*Caulobacter* Lysogenic Bacteria from Soil and the Discovery of Broad-Host-Range Phages Infecting Multiple Genera

**DOI:** 10.3390/microorganisms12091894

**Published:** 2024-09-14

**Authors:** Tannaz Mohammadi, Bert Ely

**Affiliations:** Department of Biological Sciences, University of South Carolina, Columbia, SC 29208, USA; tannaz@email.sc.edu

**Keywords:** lysogenic bacteria, phage–bacteria interactions, broad-host-range bacteriophages, microbial interactions, bacterial diversity, *Caulobacter*

## Abstract

To explore how microbial interactions within the rhizosphere influence the diversity and functional roles of bacterial communities, we isolated 21 bacterial strains from soil samples collected near Rocky Branch Creek on the University of South Carolina campus. Our findings revealed that a significant proportion of the isolated bacterial strains are lysogenic. Contrary to predictions of a narrow host range, most of the bacteriophages derived from these lysogenic bacteria demonstrated the ability to infect a broad range of bacterial strains. These results suggest that the bacterial community shares a complex phage community, creating an intricate web of interactions. This study enhances our understanding of the relationships between phages and their bacterial hosts in soil ecosystems, with implications for ecological balance and agricultural practices aimed at improving plant health through microbial management strategies.

## 1. Introduction

A wide variety of bacteria are associated with plant roots. Many of these bacteria have the capacity to enhance plant growth or improve stress tolerance through diverse mechanisms. These mechanisms include hormone modulation, the facilitation of nutrient uptake, and the suppression of diseases [1,2,3]. Thus, plant-growth-promoting rhizobacteria (PGPR) [4] have garnered increasing attention due to their potential to offer sustainable and ecological solutions to agricultural challenges [5]. Recent reports suggest that *Caulobacter* species play a functional role in the plant microbiome [6,7], leading to their classification as a hub species due to their pivotal interactions with plants. This recognition highlights their importance in shaping plant health and ecosystem dynamics [8]. *Caulobacter* is a genus of Gram-negative, aerobic, oligotrophic bacteria that is widely distributed in freshwater and soil [9,10] and is part of the α-subdivision of *Proteobacteria* [11]. It is an unusual bacterium because it divides into two different cell types, swarmer cells and stalked cells, due to asymmetric cell division [9]. The mature ‘stalked cell’ is sessile and has a stalk with a terminal holdfast that is a biological adhesive that enables it to stay in one place. The immature form, known as a ‘swarmer cell’, has a single flagellum located at one pole of the cell and is motile. Upon maturation, the swarmer cell sheds its flagellum and becomes a mature cell as it grows a stalk to replace the flagellum. This mature cell can then replicate its DNA and divide [9].

**Figure 1:**
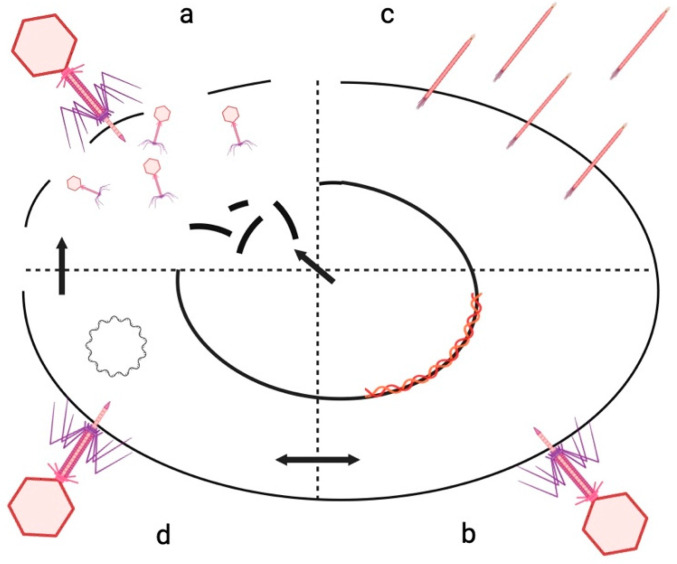
The life cycle of bacteriophages. (**a**) Lytic cycle: Bacteriophages bind to bacterial cells, inject their DNA into the bacteria, replicate the DNA and assemble it into protein capsid structures, and then lyse the cell and release new phage particles. (**b**) Lysogenic cycle: The phage DNA genome integrates into the host chromosome and then replicates as part of the bacterial chromosome. Under stress conditions, it is separated from the bacterial chromosome and enters the lytic cycle. (**c**) Chronic cycle: Bacteriophages do not kill their host because new phage particles are synthesized and extruded one by one from their host cells. Chronic infection is common with filamentous phages. (**d**) Pseudolysogenic cycle: The phage DNA genome exists in the bacterial cell as an episome. Depending on subsequent conditions, the phage DNA can adopt either the lytic cycle or the lysogenic cycle.

Bacteriophages, also known as phages, are viruses that infect bacteria, and they are found in almost all environments, including soil, fresh, and marine water samples, and most recently, in the free atmosphere [12,13]. Bacteriophages can have one of four different life cycles (Figure 1): lytic, lysogenic, chronic, and pseudolysogenic cycles. Typically, lytic phages bind to bacterial receptors, inject their DNA or RNA into the bacteria, replicate their genome, package it into protein capsid structures, and then lyse the cell and release new phage particles. However, during the alternative of a lysogenic infection, the phage DNA genome integrates into the host chromosome and then replicates as part of the bacterial chromosome which is inherited by the resulting daughter cells. This chromosomal integration will continue until stress conditions, such as DNA damage, cause the lysogenic phage genome to excise from the bacterial chromosome and enter the lytic cycle. The newly formed phage particles are then released through host–cell lysis and can infect another host bacterium. Functionally, a lysogenic pattern is one that enhances both phage and host survival, particularly under adverse conditions since lysogeny generally protects the host–cell from infection by that type of phage [14,15]. In the chronic cycle, phages do not kill their host because new phage particles are synthesized and extruded one by one from their host cells. Chronic infection is common with filamentous phages [16]. Finally, in the pseudolysogenic cycle, the bacteriophage genome is unstable in a host bacterium such that the phages neither lyse the host nor integrate into the host chromosome, and instead, they reside within the cell in a plasmid-like form called an episome [17,18]. Under starvation conditions, there is not enough energy for the phage to initiate either the lysogenic cycle or the lytic cycle. When the host–cell’s nutrient level increases, the phage is energized to integrate into the host’s chromosome as true lysogeny or to lyse cells and release new phage particles [17].

Most well-studied model bacteriophages have a narrow host range infecting only a single species of bacteria [19,20], whereas broad-host-range phages can infect multiple species or even bacteria from different genera [21]. Although bacteriophages are abundant and widely distributed, there have been limited investigations into their diversity in natural ecosystems. Gaining knowledge about the diversity of phages present in natural environments is crucial to comprehending their interactions with bacterial communities in these ecosystems.

Both Stewart and Levin [22], and later Marsh and Wellington [23], suggested that the heterogeneous nature of soil leads to a patchy distribution of host bacteria and diversity factors that should favor the increased survival of temperate phages. To test this idea, we have isolated seven new *Caulobacter* strains and fourteen new non-*Caulobacter* strains from a soil sampling site next to Rocky Branch Creek on the campus of the University of South Carolina. Our results have shown that a high percentage of both the *Caulobacter* and the non-*Caulobacter* soil strains that we sampled are lysogenic bacteria. However, in contrast to the narrow host range prediction, we found that most of the bacteriophages purified from these lysogenic bacteria can infect both *Caulobacter* and non-*Caulobacter* strains.

## 2. Materials and Methods

### 2.1. Bacterial Strain Isolation and Growth

To isolate additional *Caulobacter* strains, soil and plant roots obtained from our sampling site on the bank of Rocky Branch Creek on the University of South Carolina campus were soaked for 24 h in room-temperature sterilized tap water to allow the release of bacteria. Subsequently, the suspension was passed through a 0.45 µm filter. After the filtration step, soil particles on a filter were streaked on a PYE plate [24] containing ampicillin (20 mg/L) since we have shown that most *Caulobacter* wild-type strains are ampicillin-resistant. The plates were incubated at 30 °C. After two nights of incubation, single colonies were chosen and streaked twice on PYE ampicillin plates to isolate individual single colonies. The resulting strains were observed under a light microscope, and colonies from different bacterial plates with characteristics of *Caulobacter* strains were selected and suspended in 3 mL of PYE broth [24]. After an overnight incubation at 30 °C, bacterial DNA was extracted from each culture using a Qiagen DNA isolation kit (Qiagen GmbH 40724 Hilden, Germany). Subsequently, each bacterial DNA sample underwent PCR amplification using forward and reverse primers targeting the 16S rDNA region. The resulting 16S rDNA sequences were then compared to known bacterial 16S rDNA sequences via NCBI BLASTn analysis to determine the genus of each isolate.

### 2.2. Lysogeny Test

First, 10 μL of an overnight culture of each bacterium was carefully spotted onto PYE plates overlaid with 100 μL of either *Caulobacter vibrioides* CB15 or CB13 cultures in 3.5 mL of SSM (PYE plus 0.3% agar) on PYE agar plates. Following overnight incubation at 34 °C, the plates were examined for the presence of lysis around the spotted bacterial growth.

If lysis was observed, 500 µL from each bacterial culture was transferred into a 250 mL flask containing 25 mL of PYE broth and incubated in a shaker at 30 °C overnight. The following day, the bacterial cultures were centrifuged at least twice at 7000× *g* for 10 min to pellet the bacterial cells. Once no pellets were observed after centrifugation, 1 mL of chloroform was added to each of the supernatants, and the mixtures were shaken thoroughly to ensure the complete removal of bacteria. Subsequently, 10 μL of each sample was carefully spotted onto bacterial lawns, using the same procedure as before. After overnight incubation at 34 °C, the plates were examined for the presence of plaques or lysis, which would suggest the presence of phages. If lysis was observed, the new phage was purified twice from a single plaque. The phage titer was determined by plating serial dilutions on lawns of the host strain in SSM. In addition, 500 μL aliquots of the lysate were frozen at −70 °C for long-term storage.

### 2.3. Lysogenic Phage Induction with Mitomycin C

A volume of 500 µL from each bacterial culture was added to a 250 mL flask containing 25 mL of PYE broth and incubated in a shaker at 30 °C until the cultures reached the log phase growth. To induce lysogenic phages, 24 μL of a Mitomycin C solution (12.5 mg/mL) was added to flasks containing 25 mL of log phase bacterial cultures. The cultures were then incubated overnight in a shaker at 30 °C. The following day, the bacterial cultures were centrifuged at 7000× *g* for 10 min to pellet the bacterial cells. Subsequently, 1 mL of chloroform was added to each of the supernatants, and the mixtures were shaken thoroughly to ensure the complete removal of bacteria. Dilutions of the lysates were then mixed with 100 μL of the CB15 or CB13 host bacteria in 3.5 mL of SSM and layered on PYE plates. After overnight incubation, isolated plaques were chosen for further purification.

Additionally, for bacterial strains such as RBW1, RBW6, RBW11, RBW18, RBW23, and RBW25, which did not exhibit lytic activity against *Caulobacter* strains CB15 or CB13, 10 μL of the resultant supernatant from each sample was carefully spotted onto lawns of 21 recently isolated bacterial strains. Following an overnight incubation at 34 °C, the plates were examined for the formation of plaques or signs of lysis, indicating potential lytic activity against these strains.

### 2.4. Host Range Determination

Host range was determined by mixing 100 μL of a potential host culture with 3.5 mL of melted PYE SSM and pouring the mixture onto the surface of a PYE plate. After the top layer cooled and solidified, a 10 μL aliquot of each phage lysate was spotted on the surface. After overnight incubation at 34 °C, each spot was examined for the ability to produce a clear zone indicating that the phage could lyse the potential host cells. The host strains tested were *C. vibrioides* strains CB15, CB2, CB13, RBW21, RBW22, and RBW29; *C. segnis* strains CBR1 and TK0059; FWC26 and ME4 that belong to a third un-named *Caulobacter* species; *Rhizosphaerae* strains RBW14, RBW23, RBW25, and RBW26; *Sphingomonas* strains RBW1 and RBW11; *Acidovorax* strains RBW12 and RBW13; *Variovorax paradoxus* strain RBW16; *Xanthomonas* strains RBW17 and RBW19; *Brevundimonas* strain RBW18; *Pseudomonas* strains RBW20 and RBW6; *Bacillus cereus* strain RBW27; *Chromobacterium* strain RBW28; *Lysobacter firmicutimachus* strain RBW3; and *Rhizobium* strain RBW7.

## 3. Results

### 3.1. Isolation and Characterization of Bacterial Strains from Soil and Plant Roots

A total of 21 bacterial strains (RBW designations) were isolated from soil and plant roots collected along Rocky Branch Creek at the University of South Carolina. The primary objective of this isolation effort was to target Caulobacter bacteria, known for their resilience to ampicillin and slow growth characteristics, requiring a minimum of 2 days to develop on PYE agar supplemented with ampicillin.

To identify the newly isolated strains, the 16S rDNA gene from each bacterial isolate was amplified using PCR. Subsequently, the nucleotide sequences obtained from the PCR products were analyzed to determine the genus of each strain. Among the 21 bacterial strains isolated, 7 were identified as members of the genus Caulobacter (Table 1). To further characterize the Caulobacter strains, PCR primers designed to amplify the CB15 dnaK gene were used to generate PCR products from each strain for Sanger sequencing. The nucleotide sequence results indicated that four strains, RBW14, RBW23, RBW25, and RBW26, were most closely related to *C. rhizosphaerae*, and three strains, RBW21, RBW22, and RBW29, were most closely related to *C. vibrioides*.

The other fourteen strains included two *Sphingomonas* strains (RBW1 and RBW11), two *Acidovorax* strains (RBW12 and RBW13), a *Variovorax* strain (RBW16), two *Xanthomonas* strains (RBW17 and RBW19), a *Brevundimonas* strain (RBW18), two *Pseudomonas* strains (RBW20 and RBW6), a *Bacillus* strain (RBW27), a *Chromobacterium* strain (RBW28), a *Lysobacter* strain (RBW3), and a *Rhizobium* strain (RBW7).

### 3.2. Detection of Bacteriophages Capable of Lysing Caulobacter Strains

When streaks of each of the 21 bacterial strains were replicated onto lawns of *Caulobacter* strains CB15 and CB13, four strains, *Pseudomonas* strain RBW20, *Bacillus cereus* strain RBW27, *Chromobacterium* strain RBW28, and *Caulobacter vibrioides* strain RBW29, lysed strain CB13. However, only RBW27 and RBW29 were able to lyse strain CB15 as well. Thus, bacteriophages associated with both Gram-negative and Gram-positive strains were able to infect our laboratory *Caulobacter* host strains.

To determine whether any other strains harbored complete bacteriophage genomes, cultures of each strain were grown in the presence of mitomycin C, and phages that could lyse either CB15 or CB13 were obtained from 15 strains (Table 2; phages have RBC designations). The resulting eighteen bacteriophages obtained from the lysogenic bacteria included five from *Caulobacter* strains, RBW14, RBW21, RBW22, RBW26, and RBW29 (Table 2). In addition, 10 prophages capable of infecting CB15 or CB13 were isolated from *Acidovorax* strains RBW12 and RBW13, *Variovorax* strain RBW16, Xanthomonas strains RBW17 and RBW19, *Pseudomonas* strain RBW20, *Lysobacter* strain RBW3, *Rhizobium* strain RBW7, a *Bacillus cereus* strain (RBW27), and *Chromobacterium* strain RBW28 (Table 2). These results demonstrate that most of these phages are capable of infecting multiple bacterial genera.

Since 15 of the 21 bacterial strains isolated in this study were lysogenic for phages capable of infecting the CB15 or CB13 *Caulobacter* strains, we tested supernatants from Mitomycin C-treated cultures of the remaining six bacterial strains against all twenty-one of the bacterial strains isolated in this study. Four of the six strains, *Sphingomonas* strain RBW1, *Pseudomonas* strain RBW6, *Brevundimonas* strain RBW18, and *Caulobacter rhizosphaerae* strain RBW23, were able to lyse other bacterial strains (Table 3). Only two of the strains, RBW11 and RBW25, showed no infectivity towards any of the tested strains, suggesting that they may be non-lysogenic bacteria. Thus, all but two of the twenty-one bacterial strains harbored a lysogenic bacteriophage genome that could form a lytic broad-host-range bacteriophage.

### 3.3. Characterization of Bacteriophage Host Ranges

To better characterize the broad-host-range of our newly discovered bacteriophages, we tested the phages on all the new rhizosphere bacterial strains. These phages demonstrated infectivity not only towards *Caulobacter* laboratory strains (CB15, CB13, CB2, CBR1, FWC26, and TK0059), but also towards recently isolated soil Caulobacter strains (RBW14, RBW23, RBW26), which were closely related to *C. rhizosphaerae*, and RBW22, closely related to *C. vibrioides*. Remarkably, their infectivity extended beyond *Caulobacter* strains to include non-*Caulobacter* strains such as *Sphingomonas frigidaeris* (RBW1) and *Acidovorax* strains (RBW12 and RBW13) (Table 4). Also, although most prophages are thought to protect their host strains from infection by closely related bacteriophages, we found that six of the nineteen prophages can infect their original lysogenic host strains (Table 3 and Table 4).

## 4. Discussion

The isolation and characterization of bacterial strains from soil and plant roots along Rocky Branch Creek at the University of South Carolina provided a snapshot of the role of Caulobacters thriving in this unique environment. We isolated 21 bacterial strains including seven *Caulobacter* strains and fourteen additional bacteria that share some properties with Caulobacters. The non-*Caulobacter* strains included representatives of the *Sphingomonas, Acidovorax, Variovorax, Xanthomonas, Brevundimonas, Pseudomonas, Bacillus, Chromobacterium, Lysobacter, and Rhizobium* genera.

One notable finding from this study was the discovery that 19 out of 21 bacterial isolates harbored bacteriophages with broad host ranges, and 6 of these strains were capable of being re-infected by the phages they harbor as lysogens. Surprisingly, these lysogenic bacteria were not protected by their resident phage genomes and remained susceptible to various additional bacteriophages, such as the new genus of *Dolichocephalovirinae*.

This broad host range of the phages highlights the complex interactions within the microbial ecosystem. The ability of these phages to infect a diverse array of bacterial hosts suggests a dynamic and interconnected phage–bacteria network, which likely plays a significant role in shaping microbial community structure and function. Understanding these intricate phage–host relationships is crucial for elucidating the roles of bacteriophages in microbial ecology. For example, the broad-host-range phages likely play a role in the composition of the bacterial rhizosphere community. Moreover, these insights have potential applications in biotechnology, agriculture, and environmental management, where phages could be leveraged for controlling bacterial populations and enhancing ecosystem health. These dynamics may reveal novel aspects of phage biology and their impact on ecosystem resilience and functionality.

Future research could focus on identifying the molecular mechanisms behind phage–host interactions and the genetic determinants of host specificity. Additionally, examining the genetic information of newly discovered prophages could reveal novel species, genera, or even families of viruses. A deeper understanding of these dynamics could provide valuable insights into the evolutionary processes of phages and bacteria. This knowledge has the potential to advance various applications in biotechnology, including phage therapy and biocontrol strategies.

## Figures and Tables

**Table 1:** Colony characteristics of 21 bacteria isolated from soil and plant roots along Rocky Branch Creek.

Bacterial Name	Bacterial Strain	Colony Size	Pigmentation	Form	Elevation	Margin
RBW1	*Sphingomonas frigidaeris*	Pinpoint	Yellow	Circular	Convex	Entire
RBW3	*Lysobacter*	Large	Yellow	Irregular	Umbonate	Undulate
RBW6	*Pseudomonous*	Large	White	Irregular	Flat	Undulate
RBW7	*Rhizobium*	Large	White	Circular	Pulvinate	Entire
RBW11	*Sphingomonas* sp.	Pinpoint	Yellow	Circular	Convex	Entire
RBW12	*Acidovorax* sp.	Medium	White	Circular	Convex	Entire
RBW13	*Acidovorax* sp.	Medium	White	Circular	Umbonate	Erose
RBW14	*Caulobacter rhizosphaerae*	Large	White	Irregular	Umbonate	Undulate
RBW16	*Variovorax*	Large	Yellow	Irregular	Umbonate	Undulate
RBW17	*Xanthomonas translucens*	Large	Yellow	Irregular	Umbonate	Undulate
RBW18	*Brevundimonas*	Large	Orange	Circular	Umbonate	Entire
RBW19	*Xanthomonas*	Pinpoint	Yellow	Circular	Convex	Entire
RBW20	*Pseudomonous*	Large	White	Irregular	Umbonate	Undulate
RBW21	*Caulobacter vibrioides*	Large	White	Irregular	Umbonate	Undulate
RBW22	*Caulobacter vibrioides*	Large	White	Circular	Umbonate	Undulate
RBW23	*Caulobacter rhizosphaerae*	Large	Yellow	Irregular	Umbonate	Undulate
RBW25	*Caulobacter rhizosphaerae*	Medium	Yellow	Circular	Convex	Entire
RBW26	*Caulobacter soil strain*	Small	Yellow	Circular	Flat	Entire
RBW27	*Bacillus cereus*	Large	White	Rhizoid	Umbonate	Lobate
RBW28	*Chromobacterium* sp.	Large	White	Irregular	Umbonate	Undulate
RBW29	*Caulobacter vibrioides*	Small	White	Circular	Convex	Entire

**Table 2:** Identification of lysogenic bacterial strains capable of infecting CB15 or CB13 *Caulobacter* strains.

Lysogenic Bacteria	CB15	CB13
RBW1	−	−
RBW3	−	+
RBW6	−	−
RBW7	−	+
RBW11	−	−
RBW12	+	−
RBW13	+	−
RBW14	−	+
RBW16	+	−
RBW17	+	−
RBW18	−	−
RBW19	+	−
RBW20	−	+
RBW21	+	+
RBW22	+	−
RBW23	−	−
RBW25	−	−
RBW26	+	−
RBW27	+	−
RBW28	−	+
RBW29	−	+

**Table 3:** Identification of 4 additional lysogenic bacterial strains.

RBWs Bacteria	RBW1	RBW6	RBW11	RBW18	RBW23	RBW25
RBW1	+	−	−	−	−	−
RBW3	−	−	−	−	−	−
RBW6	−	−	−	−	−	−
RBW7	−	−	−	−	−	−
RBW11	−	−	−	−	−	−
RBW12	−	−	−	−	−	−
RBW13	+	−	−	−	+	−
RBW14	−	−	−	−	−	−
RBW16	−	−	−	−	−	−
RBW17	−	−	−	−	−	−
RBW18	−	−	−	−	−	−
RBW19	+	+	−	+	+	−
RBW20	+	−	−	−	+	−
RBW21	+	+	−	+	+	−
RBW22	−	+	−	−	+	−
RBW23	−	+	−	−	+	−
RBW25	−	−	−	−	−	−
RBW26	−	−	−	−	−	−
RBW27	+	+	−	+	+	−
RBW28	−	−	−	−	−	−
RBW29	−	−	−	−	−	−

**Table 4:** Bacteriophage host range and self-sensitivity.

Bacteriophage Name	Original Host	Bacterial Strain	Plaque Morphology ^1^	Positive Host Range	Self
RBC51	RBW3 *Lysobacter*	CB13	Turbid	CB15, CB13, CB2, RBW1, RBW14, RBW23	−
RBC52	RBW7 *Rhizobium*	CB13	Turbid	CB15, CB13, CB2, RBW1, RBW13, RBW14, RBW22	−
RBC53	RBW12 *Acidovorax*	SC1004	Turbid	CB15, CB13, CB2, FWC26, RBW1, RBW12, RBW14, RBW22, RBW23	+
RBC54	RBW12 *Acidovorax*	SC1004	Clear	CB15, CB13, CB2, FWC26, RBW1, RBW12, RBW22, RBW23	+
RBC55	RBW13 *Acidovorax*	SC1004	Turbid	CB15, CB13, CB2, CBR1, RBW14, RBW22	−
RBC56	RBW14 *Caulobacter rhizosphaerae*	CB13	Turbid	CB15, CB13, CB2, FWC26, CBR1, RBW14, RBW12	+
RBC57	RBW16 *Variovorax*	SC1004	Clear	CB15, CB13, CB2, CBR1, FWC26, TK0059, RBW12, RBW14, RBW22, RBW23, RBW26	−
RBC58	RBW16 *Variovorax*	SC1004	Turbid	CB15, CB13, CB2, RBW12, RBW14, RBW22	−
RBC59	RBW17 *Xanthomonas*	SC1004	Clear	CB15, CB13, CB2, TK0059, RBW12, RBW14, RBW22	−
RBC60	RBW19 *Xanthomonas*	SC1004	Clear	CB15, CB13, CB2, CBR1, TK0059, RBW12, RBW14, RBW22, RBW23	−
RBC61	RBW20 *Pseudomonas*	SC1004	Clear	CB15, CB13, CB2, RBW12, RBW14	−
RBC62	RBW21 *Caulobacter vibrioides*	SC1004	Clear	CB15, CB13, CB2, RBW14	−
RBC63	RBW21 *Caulobacter vibrioides*	CB13	Clear	CB15, CB13, CB2, FWC26, RBW12, RBW13, RBW14, RBW22	−
RBC64	RBW22 *Caulobacter vibrioides*	SC1004	Clear	CB15, CB13, CB2, FWC26, RBW13, RBW14, RBW22	+
RBC65	RBW27 *Bacillus cereus*	SC1004	Clear	CB15, CB13, CB2, RBW12, RBW13, RBW14, RBW22	−
RBC66	RBW28 *Chromobacterium*	CB13	Turbid	CB15, CB13, CB2, FWC26, RBW1, RBW12, RBW13, RBW14, RBW22, RBW23	−

^1^ Turbid plaques are caused by incomplete lysis of the host cells.

## Data Availability

The original contributions presented in the study are included in the article, further inquiries can be directed to the corresponding author.
